# Terminal Ileal Ulcers: Endoscopic Predictors for the Later Development of Crohn’s Disease

**DOI:** 10.3390/diagnostics16091339

**Published:** 2026-04-29

**Authors:** Weiqi Zhang, Kun Zhang, Yang Yang, Haibin Dong, Shan Xie, Xiaojuan Guo, Bo Jiang, Yutang Ren, Shiming Zhou

**Affiliations:** Department of Gastroenterology, Beijing Tsinghua Changgung Hospital, School of Clinical Medicine, Tsinghua Medicine, Tsinghua University, Beijing 102218, Chinayyang_tmmu@163.com (Y.Y.);

**Keywords:** terminal ileal ulcers, Crohn’s disease, surgical risk

## Abstract

**Background:** Terminal ileal ulcers (TIUs) are frequently identified during colonoscopy but are often attributed to nonspecific etiologies, potentially delaying the diagnosis of Crohn’s disease (CD). This study aimed to evaluate the utility of TIU as an early diagnostic marker and to determine whether diagnosis at the isolated TIU stage impacts long-term surgical outcomes. **Methods:** In this single-center, retrospective cohort study, consecutive adult patients with TIU detected via colonoscopy over a 10-year period were analyzed. Patients were stratified into CD-TIU and non-CD-TIU cohorts based on longitudinal follow-up. Clinical, endoscopic, and laboratory parameters were compared. Long-term surgical outcomes of patients diagnosed at the isolated TIU stage (Montreal classification L1) were compared with those of patients with established ileocolonic disease (Montreal classification L3). **Results:** Of the 66 patients included in the final analysis, 18 (27.3%) were ultimately diagnosed with CD. Specific endoscopic features—including longitudinal or fissuring ulcers (50.0% vs. 6.3%, *p* < 0.001), ileocecal valve deformity (50.0% vs. 18.8%, *p* = 0.011), and increased ulcer dimensions (9.11 mm vs. 4.60 mm, *p* = 0.002)—were significantly associated with a CD diagnosis. Notably, patients diagnosed at the TIU stage (CD-L1) exhibited a significantly lower surgical rate compared to those with established ileocolonic disease (CD-L3) (5.6% vs. 70.2%, *p* < 0.001). **Conclusions:** TIU, particularly when characterized by longitudinal morphology or ileocecal valve involvement, constitutes a critical early manifestation of CD. Diagnosis at the isolated TIU stage is associated with a lower long-term surgical risk compared to established ileocolonic disease, likely reflecting the influence of disease phenotype. These findings underscore the imperative for meticulous endoscopic evaluation to identify early-stage disease.

## 1. Introduction

Crohn’s disease (CD), a chronic and progressive subtype of inflammatory bowel disease (IBD), exhibits a global incidence comparable to that of ulcerative colitis, yet imposes a significantly higher burden of complications [1]. Driven by rising incidence in industrialized regions [2], CD has contributed to IBD now affecting over 0.3% of the Western population [3]. Projections indicate that the overall prevalence of IBD will increase from 660 to 790 cases per 100,000 population between 2015 and 2025, with observed rates likely to surpass these estimates [4]. Despite this escalating epidemiological trend, the diagnostic paradigm for CD remains suboptimal; the absence of validated biomarkers for early detection necessitates reliance on a multimodal assessment integrating clinical, serological, fecal, endoscopic, and histological findings [5,6]. Consequently, a substantial proportion of patients are diagnosed only after developing stricturing or penetrating complications requiring surgical intervention [7]. This diagnostic delay directly exacerbates the profound economic burden of CD, which is primarily driven by direct costs (approximately €3500 per patient annually) stemming from recurrent surgeries and biologic therapies, alongside indirect costs (approximately €1900 per patient annually) related to disability and occupational impairment [8,9,10,11]. These converging challenges underscore an urgent need for identifiable early-disease signatures to mitigate preventable morbidity.

The terminal ileum (TI) plays a pivotal role in the pathogenesis of CD, acting as a critical site for early lesion formation owing to its distinct anatomical, genetic, microbial, and immunological characteristics [12]. Anatomically, the high density of Paneth cells and Peyer’s patches maintains mucosal barrier integrity via antimicrobial peptide secretion and immune surveillance, functions that are compromised during the early phases of CD [13,14,15,16,17,18]. Genetically, risk variants in *NOD2* [17,19,20,21] and *ATG16L1* [13,14,16,17,22] specifically impair ileal bacterial sensing and Paneth cell autophagy, precipitating endoplasmic reticulum stress prior to the onset of macroscopic inflammation [23]. Ileal dysbiosis, characterized by the early depletion of *Faecalibacterium prausnitzii* [24,25] and the expansion of adherent invasive *Escherichia coli* [24,25,26], facilitates epithelial breach via the upregulation of CEACAM6 receptors [27,28]. Immunologically, Th1/Th17 polarization and the formation of mesenteric creeping fat originate within this region [29,30,31,32]. Crucially, these interconnected pathological perturbations precede the development of overt inflammation, positioning terminal ileitis as potentially the earliest clinical manifestation of CD. Consequently, endoscopic features identified prior to a definitive CD diagnosis serve as early markers, while isolated terminal ileal involvement (Montreal L1, B1) represents disease in its early stage.

Colonoscopy remains the gold standard for evaluating colonic disorders, and TIUs are a relatively common incidental finding during such procedures. However, owing to a limited understanding of their clinical significance, TIUs are often underappreciated and readily dismissed as insignificant or nonspecific [33,34]. This oversight precludes the opportunity for the early identification of underlying pathological conditions, despite the fact that most TIUs possess specific etiologies [35,36,37] for which timely diagnosis and appropriate management could prevent severe complications. Notably, persistent TIU is strongly associated with the subsequent development of CD [38,39,40,41]. Given that early diagnosis is paramount for improving the prognosis of CD, elucidating the clinical significance of TIU is critical for optimizing diagnostic strategies and improving patient outcomes.

To address these critical gaps, this real-world study has dual objectives: first, to systematically identify specific endoscopic predictors of CD in patients presenting with TIU; second, to evaluate whether early diagnosis and intervention at the isolated TIU stage (CD-L1) confer a long-term prognostic benefit by reducing surgical risk compared to established ileocolonic disease (CD-L3).

## 2. Materials and Methods

### 2.1. Study Design and Setting

This retrospective, real-world, observational cohort study was conducted at the Digestive Disease Center, Beijing Tsinghua Changgung Hospital, Tsinghua University. The study period extended from 1 January 2016, to 31 December 2025. Owing to the retrospective design, the Institutional Review Board waived the requirements for both ethical approval and informed consent. The diagnosis of CD was established in accordance with the ECCO guidelines [6,42]. For the purposes of this study, “early-stage CD” was defined as newly diagnosed disease strictly confined to the terminal ileum with an inflammatory phenotype at index colonoscopy, representing the earliest clinical manifestation of the disease spectrum.

### 2.2. Study Population

All consecutive adult patients (aged ≥ 18 years) who underwent colonoscopy with TI intubation during the study period were screened.

Inclusion Criteria: Patients were included if their endoscopic reports explicitly documented the presence of “ulcer(s)” or “ulceration” in the TI. An ulcer was defined as a mucosal break with discernible depth, extending beyond superficial erosion. To ensure diagnostic consistency, this definition was corroborated against available endoscopic images and medical record descriptions for all included patients.

Exclusion Criteria: (i) A pre-existing diagnosis of IBD (including CD, ulcerative colitis, or IBD-unclassified) prior to the index colonoscopy. (ii) Identifiable alternative etiologies for TIU. Specifically, infectious etiologies were rigorously ruled out through negative stool cultures (including *Salmonella*, *Shigella*, *Campylobacter*, and *Yersinia*) and the absence of cytomegalovirus inclusions on histopathology. NSAID-induced ulceration was excluded based on a documented absence of NSAID or aspirin use within four weeks prior to the index colonoscopy. This stringent exclusion of transient or drug-induced causes ensured that the remaining TIU cases were more likely representative of chronic, underlying pathology, such as early CD. (iii) Inadequate follow-up data, defined as the availability of only a single colonoscopy report without subsequent clinical, laboratory, or endoscopic follow-up.

### 2.3. Statistical Analysis

All statistical analyses were performed using GraphPad Prism version 7 (GraphPad Software Inc., San Diego, CA, USA) and SPSS Statistics version 27 (IBM Corp, Armonk, NY, USA). Categorical variables were compared between groups using the χ^2^ test, and results were presented in three line tables. Continuous data are expressed as mean ± standard deviation (SD), whereas categorical data are presented as frequencies or percentages. A two-sided *p* value of less than 0.05 was considered statistically significant.

Time zero for this study was set as the date of a patient’s first colonoscopy performed at our institution. The observation period began from this date and continued until the earliest occurrence of any of the following events: (1) loss to follow-up, defined as failure to complete two or more colonoscopy examinations at our hospital; (2) among patients who underwent two or more colonoscopies, having only one examination showing terminal ileal ulcers; (3) death from causes unrelated to the outcome of interest; or (4) the study cut-off date (31 December 2025). Based on a review of the literature and clinical judgment, we prespecified several variables for inclusion in the multivariate Cox regression model: age, sex, and treatment regimen. Given the limited sample size, only variables with a univariate analysis *p* value < 0.05 and known strong confounders (e.g., age and sex) were entered into the multivariate model.

To assess surgical risk, a Cox proportional hazards regression model was employed. Given the limited sample size of the CD-TIU group (*n* = 18), the model was restricted to the most clinically relevant variables (age, sex, and treatment modality) to prevent overfitting. Disease phenotype (L1 versus L3) served as the primary grouping variable for the comparative surgical analysis.

Endoscopic predictors of CD diagnosis were evaluated via univariate analyses (the χ^2^ test for categorical variables and the Student *t* test for continuous variables). To associate the early endoscopic stage (represented by the L1 group) with surgical outcomes over the long term, a time to event analysis was conducted using the Cox proportional hazards regression model. This survival analysis was selected to appropriately account for varying follow-up durations and the temporally dependent nature of surgical interventions. The model was adjusted for sex, age at diagnosis, and treatment modality.

## 3. Results

### 3.1. Baseline Characteristics

**Figure 1:**
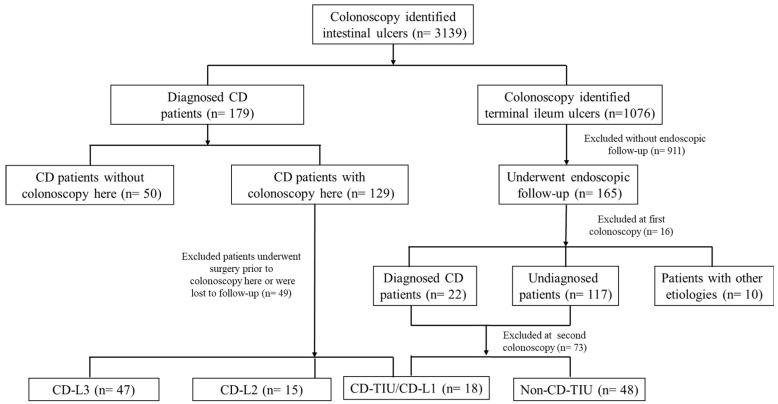
Flowchart of patient screening and inclusion.

During the study period, colonoscopy identified 3139 patients with intestinal ulcers, including 1076 with ulcers localized specifically to the terminal ileum. After excluding patients with prior IBD, confirmed alternative etiologies, or insufficient follow-up data, A total of 165 patients with TIU were available for longitudinal assessment via repeat colonoscopy. Subsequent exclusion of patients with self-resolving, non-specific etiologies yielded a final study cohort of 66 patients with TIU (Figure 1).

Among these 66 patients, 18 (27.3%) were ultimately diagnosed with Crohn’s disease (CD-TIU group), while 48 (72.7%) had non-CD etiologies (non-CD-TIU group). Patients in the CD-TIU group were numerically younger (40.33 ± 16.87 years) compared to the non-CD-TIU group (47.90 ± 13.08 years), though this difference was not statistically significant (*p* = 0.058). Endoscopic follow-up was significantly longer in the non-CD-TIU group (64.06 ± 26.10 months) than in the CD-TIU group (41.22 ± 28.82 months, *p* = 0.003). There were no significant differences in sex distribution between the two groups. Laboratory parameters, including white blood cell count, hemoglobin, platelet count, and albumin levels, did not significantly differ between groups (Table 1).

### 3.2. Endoscopic Features

**Figure 2:**
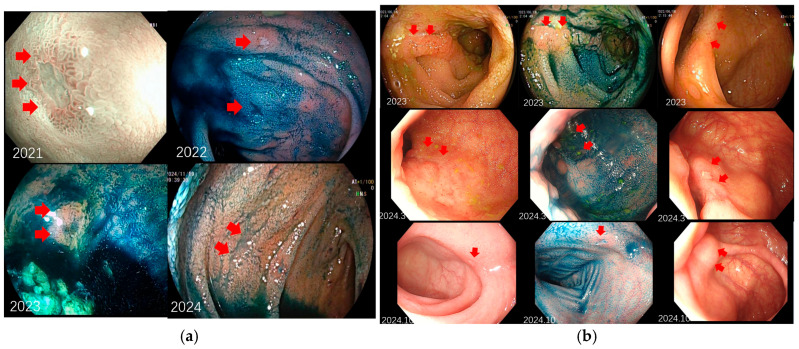
(**a**) Dynamic progression of TIU with gradual development of longitudinal ulceration; (**b**) Progressive involvement of the ileocecal valve by advancing ulceration. Red arrows denote lesions. Numbers denote the year and month of follow-up (e.g., October 2024).

The ulcers in CD-TIU demonstrated dynamic evolution during endoscopic follow-up, whereas no such changes were observed in the non-CD TIU group (Figure 2). Ulcer morphology was strongly associated with CD diagnosis (*p* < 0.001): longitudinal or fissuring ulcers were significantly more common in the CD-TIU group (50.0%, *n* = 9) compared to the non-CD TIU group (6.3%, *n* = 3). In contrast, punctate ulcers were exclusively observed in the non-CD group (50.0%, *n* = 24). Ulcer size was significantly larger in the CD-TIU group (9.11 ± 11.08 mm) than in the non-CD group (4.60 ± 4.54 mm, *p* = 0.002). Deformity or ulceration of the ileocecal valve was also more frequent in the CD-TIU group (50.0% vs. 18.8%, *p* = 0.011). No significant differences were observed in ulcer number, margin appearance, ulcer base, or peri-ulcer mucosal changes (Table 2).

### 3.3. Treatment Response and Long-Term Surgical Outcomes

Treatment response was evaluated during follow-up in the 18 patients diagnosed with CD-TIU. Due to the small sample size, the treatment response table is purely descriptive. At the 6-week follow-up (Table 3), 2 of the 8 patients (25.0%) treated with biologics achieved endoscopic healing, whereas none of the 8 patients receiving 5-aminosalicylates (5-ASA) achieved healing; 2 patients received no treatment. By the 52-week follow-up (Table 4), 16 patients who received treatment were included in the analysis.

To evaluate the impact of diagnosis at the TIU stage on disease progression, we compared the long-term surgical outcomes of the CD-TIU group (classified as CD-L1, isolated terminal ileal disease) with a historical cohort of patients diagnosed with established ileocolonic CD (CD-L3). This comparison is critical because CD-L3 represents the advanced disease phenotype that isolated TIU (L1) may progress to if left untreated.

**Figure 3:**
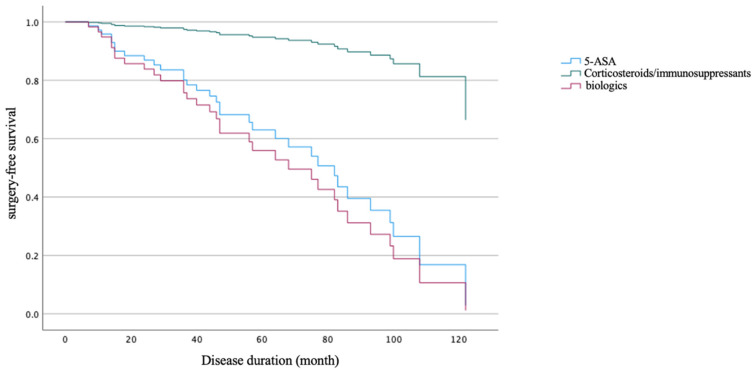
Survival function at the mean of covariates.

The surgical risk was significantly lower in the CD-L1 group compared to the CD-L3 group (1/18 vs. 33/47; 5.6% vs. 70.2%, *p* < 0.001) (Table 5). Cox regression analysis indicated that disease location (L1 vs. L3) was associated with a trend toward lower surgical risk (Figure 3), though this did not reach statistical significance (HR = 0.149, 95% CI: 0.019–1.168, *p* = 0.070). Regarding treatment modalities, compared to biologic therapy, the use of 5-ASA was associated with a significantly lower surgical risk (HR = 0.093, 95% CI: 0.012–0.713, *p* = 0.022), whereas no significant difference was observed between 5-ASA and corticosteroids/immunosuppressants (HR = 0.796, 95% CI: 0.231–2.740, *p* = 0.718) (Table 6). However, the apparent protective effect of 5-ASA over biologics likely reflects indication bias, as biologic agents were typically reserved for patients with more severe or refractory baseline disease, whereas 5-ASA was predominantly used in milder cases.

## 4. Discussion

TIUs are frequently encountered during colonoscopy but are often clinically overlooked due to their classification as non-specific findings, posing a risk of missed CD diagnoses. This real-world study demonstrates that TIUs are not merely incidental findings but rather represent a crucial opportunity for early CD detection. We found that 27.3% of patients with TIU were ultimately diagnosed with CD, aligning with previous reports indicating that approximately one-third of such patients receive a CD diagnosis. These findings underscore the imperative for vigilant assessment of any mucosal breaks within the terminal ileum.

Longitudinal morphology, larger ulcer size, and ileocecal valve deformity were identified as notable endoscopic features associated with a subsequent CD diagnosis. The prolonged endoscopic follow-up observed in the non-CD-TIU group, compared with the CD-TIU group, likely reflects verification bias. Specifically, the dynamic evolution and distinct morphological features of ulcers in the CD-TIU cohort prompted early definitive diagnosis and treatment, thereby shortening the observation period. Conversely, the unremarkable or static nature of ulcers in non-CD-TIU patients necessitated prolonged surveillance to exclude latent pathology.

It is crucial to contextualize the actual risk of progressing to CD following a TIU diagnosis. Although 27.3% of patients in our final study cohort were diagnosed with CD, the risk of progression is substantially lower when considering the broader initial cohort of 1076 patients with TIU, or the 165 patients who underwent repeat colonoscopy. However, our data suggest that heightened clinical vigilance is warranted for TIU exhibiting longitudinal morphology, larger size, or ileocecal valve deformity. When these features are encountered, clinicians should obtain targeted biopsies for histopathological evaluation and initiate biomarker profiling (e.g., fecal calprotectin, CRP). Importantly, we do not advocate initiating CD-specific medical therapy, such as immunomodulators or biologic therapy, based solely on endoscopic findings without histological confirmation of CD. Implementing a top-down or early intensive strategy at this pre-complication stage (CD-L1) may control the inflammatory burden and prevent irreversible structural damage. The lower surgical rate observed in the CD-L1 group compared to the CD-L3 group aligns with this hypothesis, though we emphasize this reflects an observational association with disease phenotype rather than a proven causal effect of early intervention.

A pivotal finding of this study is the significant difference in surgical rates between patients diagnosed at the TIU stage and those with established ileocolonic disease. The mean follow-up duration of approximately 3.5 years in the CD-L1 group and 5.3 years in the CD-L3 group provides a sufficient window to capture meaningful surgical outcomes. Evidence suggests that the highest risk for early surgery in CD occurs within the first few years following diagnosis, particularly once penetrating or stricturing complications develop. Therefore, the significantly lower surgical rate observed in the CD-L1 group underscores the prognostic implications of disease phenotype at diagnosis. However, it cannot be concluded that early detection alone causally reduces surgical risk, as patients presenting at the L1 stage may inherently possess a less aggressive disease course.

Comparing patients with TIU to “healthy” controls or those without TIU presents inherent methodological challenges; therefore, our comparative analysis focused on disease phenotypes, contrasting the isolated L1 phenotype (detected via TIU) against the L3 phenotype (established ileocolonic disease). This comparison suggests a potential clinical advantage associated with diagnosing CD prior to progression to extensive ileocolonic involvement, although prospective studies are required to confirm whether early intervention causally improves long-term outcomes. We do not imply that the CD-L3 group experienced a “delayed diagnosis” in the context of late clinical presentation, but rather that they presented at a later, more complicated disease stage. This phenotypic comparison supports the hypothesis that identifying CD at the L1 stage provides a critical window of opportunity to alter the disease trajectory.

This study has several limitations. First, the retrospective design is inherently susceptible to selection and information biases. Notably, while 165 patients with TIU underwent repeat colonoscopy, others did not; we were unable to determine the incidence of CD in those lost to follow-up, potentially introducing selection bias. Second, the single-center design limits the generalizability of our findings. Third, while univariate analysis demonstrated a significantly lower surgical rate in the CD-L1 group compared to the CD-L3 group, the multivariate Cox regression indicated that disease location was not an independent statistically significant predictor (*p* = 0.070). The substantial difference in surgical rates inherently reflects disease phenotype as a major determinant of clinical outcomes, but the limited sample size precluded propensity score matching to fully balance confounders such as treatment indication bias. Thus, the independent effect of early diagnosis on surgical risk warrants validation in larger, prospective cohorts.

Future research should prioritize prospective multicenter cohorts to validate these findings and enhance generalizability. The clinical utility of our predictive model warrants prospective evaluation in undiagnosed TIU cohorts. Priority should be given to randomized trials assessing targeted interventions in TIU patients at elevated risk who present with longitudinal lesions, with the prevention of Crohn’s disease progression as the primary endpoint. Furthermore, integrated multidimensional biomarker panels incorporating the mucosal microbiome, host genomics, and serum proteomics may refine etiologic prediction and yield deeper mechanistic insights.

## 5. Conclusions

While the overall risk of progression to CD in patients with incidentally detected TIU is low, these findings should not be routinely dismissed as nonspecific. Specific endoscopic features, including longitudinal or fissuring morphology, increased ulcer size, and ileocecal valve deformity, should heighten clinical suspicion for underlying CD. These observations underscore the need for meticulous terminal ileal inspection, standardized reporting of ulcer characteristics, and targeted histological sampling when such features are present. Crucially, CD-specific medical therapy must not be initiated without histological confirmation. Furthermore, while a diagnosis at the isolated TIU stage (CD-L1) is associated with a lower long-term surgical risk compared to established ileocolonic disease (CD-L3), this likely reflects differences in disease phenotype rather than a causal effect of early intervention. These findings are limited by the retrospective, single-center design, a small sample size precluding advanced statistical adjustments, and the lack of standardized biopsy protocols. Future research should prioritize prospective multicenter validation of these predictors and randomized trials evaluating targeted diagnostic and therapeutic interventions in high-risk TIU patients.

## Figures and Tables

**Table 1:** Baseline and Clinical Characteristics of Study Participants Stratified by Terminal Ileal Ulcer Etiology.

	CD-TIU Group (*n* = 18)	Non-CD-TIU Group (*n* = 48)	t/χ^2^	*p*
Age at TIU diagnosis, years	40.33 ± 16.866	47.90 ± 13.075	3.760	0.058
Endoscopic Follow-up Interval, months	41.22 ± 28.819	64.06 ± 26.102	0.082	0.003
Sex			0.128	0.721
Male	10	29		
Female	8	19		
Laboratory Parameters				
WBC (×10^9^/L)	7.28 ± 3.48	6.61 ± 1.53	−0.065	0.949
HGB (g/L)	134.39 ± 21.769	145.12 ± 18.766	0.553	0.058
PLT (×10^9^/L)	291.78 ± 95.903	249.76 ± 64.523	4.312	0.052
ALB (g/L)	45.328 ± 4.587	46.603 ± 3.066	7.985	0.226

**Table 2:** Endoscopic Features of Study Participants Stratified by Terminal Ileal Ulcer Etiology.

	CD-TIU Group (*n* = 18)	Non-CD-TIU Group (*n* = 48)	t/χ^2^	*p*
Ulcer number			0.520	0.471
Single	4	15		
Multiple	14	33		
Ulcer morphology			28.169	<0.001
Round/Oval	3	16		
irregular	6	5		
Longitudinal/fissuring	9	3		
punctate	0	24		
Ulcer Margin			0.518	0.472
Flat	15	36		
Elevated	3	12		
Ulcer Base			0.091	0.763
Clean	9	26		
Slough	9	22		
Ulcer Size (mm)	9.11 ± 11.08	4.60 ± 4.54	−3.098	0.002
Peri-ulcer Mucosa			0.537	0.464
Congestion and edema	17	47		
Normal	1	1		
Ileocecal Valve Appearance			6.445	0.011
Normal	9	39		
Deformed/Ulcerated	9	9		

**Table 3:** Treatment Response at 6 w (CD-TIU Group only).

	Endoscopic Healing	Not Healing
5-ASA	0	8
Biologics	2	6
No treatment	0	2

**Table 4:** Treatment Response at 52 w (CD-TIU Group only).

	Endoscopic Healing	Not Healing
5-ASA	0	5
Biologics	1	10

**Table 5:** Treatment Response (CD-TIU Group vs. CD-L3).

	L1 Terminal Ileum (*n* = 18)	L3 Ileocolonic (*n* = 47)	t/χ^2^	*p*
			21.811	<0.001
Surgery	1	33		
No Surgery	17	14		

**Table 6:** Risk Factors for Surgery: A Cox Regression Analysis.

	Coefficient	Standard Error	Wald χ^2^	HR	95% CI	*p* Value
Gender (male = 1)	−0.406	0.378	1.151	0.666	(0.317, 1.399)	0.283
Age at diagnosis	−0.015	0.013	1.187	0.985	(0.960, 1.012)	0.276
Disease location	−1.901	1.049	3.283	0.149	(0.019, 1.168)	0.070
Treatment			5.260			0.072
5-ASA vs. corticosteroids/immunosuppressants	−0.228	0.630	0.131	0.796	(0.231, 2.740)	0.718
5-ASA vs. biologics	−2.379	1.042	5.218	0.093	(0.012, 0.713)	0.022

## Data Availability

The data presented in this study are available on request from the corresponding author. The data are not publicly available due to privacy or ethical restrictions, as they contain sensitive patient medical information that could potentially identify individuals. Access to the de-identified dataset may be granted to qualified researchers for specific research purposes upon reasonable request and approval from the Institutional Review Board of Beijing Tsinghua Changgung Hospital, Tsinghua University. Requests for data access should be directed to [zsma03637@btch.edu.cn (S.Z.)], with a clear description of the intended use and analysis plan.

## Abbreviations

The following abbreviations are used in this manuscript:

TIU	Terminal ileal ulcer
CD	Crohn’s disease
IBD	inflammatory bowel disease
